# Leveraging Tumor Microenvironment Infiltration in Pancreatic Cancer to Identify Gene Signatures Related to Prognosis and Immunotherapy Response

**DOI:** 10.3390/cancers15051442

**Published:** 2023-02-24

**Authors:** Jiabin Yang, Liangtang Zeng, Ruiwan Chen, Leyi Huang, Zhuo Wu, Min Yu, Yu Zhou, Rufu Chen

**Affiliations:** 1 School of Medicine, South China University of Technology, Guangzhou 510006, China; 2 Department of Pancreatic Surgery, Department of General Surgery, Guangdong Provincial People’s Hospital (Guangdong Academy of Medical Sciences), Southern Medical University, Guangzhou 510080, China; 3 Department of Radiation Oncology, The First Affiliated Hospital, Sun Yat-sen University, Guangzhou 510275, China; 4 Department of Pancreatobiliary Surgery, Sun Yat-sen Memorial Hospital, Sun Yat-sen University, Guangzhou 510275, China; 5 Guangdong Provincial Key Laboratory of Malignant Tumor Epigenetics and Gene Regulation Medical Research Center, Sun Yat-sen Memorial Hospital, Sun Yat-sen University, Guangzhou 510275, China

**Keywords:** pancreatic cancer, immune microenvironment, prognosis, immunotherapy, molecular subtype, F2RL1, therapeutic target

## Abstract

**Simple Summary:**

Pancreatic ductal adenocarcinoma (PDAC) has an insidious onset and rapid progression, and its morbidity and mortality are increasing year by year. Currently, there are limited therapeutic methods and no effective therapeutic guidance. Tumor microenvironments (TME) of PDAC are highly specific and associated with the failure of chemotherapy, radiotherapy, and immunotherapy. Different TMEs have different sensitivities to treatment modalities. Therefore, constructing a prediction model based on TME classification and giving corresponding treatment measures according to the classification results will provide a new idea for clinical precision diagnosis and treatment. Further verification of gene function related to TME will greatly provide effective potential clinical treatment targets for personalized therapy.

**Abstract:**

The hallmark of pancreatic ductal adenocarcinoma (PDAC) is an exuberant tumor microenvironment (TME) comprised of diverse cell types that play key roles in carcinogenesis, chemo-resistance, and immune evasion. Here, we propose a gene signature score through the characterization of cell components in TME for promoting personalized treatments and further identifying effective therapeutic targets. We identified three TME subtypes based on cell components quantified by single sample gene set enrichment analysis. A prognostic risk score model (TMEscore) was established based on TME-associated genes using a random forest algorithm and unsupervised clustering, followed by validation in immunotherapy cohorts from the GEO dataset for its performance in predicting prognosis. Importantly, TMEscore positively correlated with the expression of immunosuppressive checkpoints and negatively with the gene signature of T cells’ responses to IL2, IL15, and IL21. Subsequently, we further screened and verified F2R-like Trypsin Receptor1 (F2RL1) among the core genes related to TME, which promoted the malignant progression of PDAC and has been confirmed as a good biomarker with therapeutic potential in vitro and in vivo experiments. Taken together, we proposed a novel TMEscore for risk stratification and selection of PDAC patients in immunotherapy trials and validated effective pharmacological targets.

## 1. Introduction

Pancreatic ductal adenocarcinoma (PDAC) is one of the most challenging cancers in alimentary malignancies [1,2]. For most patients with PDAC, cytotoxic chemotherapy remains the mainstay of treatment. However, despite recent improvements in chemotherapeutic regimens and treatment modalities, their survival benefits remain limited [3]. In addition, many efforts have been made to develop targeted therapies for PDAC, but there has been no substantial improvement [4,5]. Progress in strategies targeting homologous recombination defects, while substantial, currently shows applicability and efficacy in only a small proportion of patients [6]. Furthermore, PDAC is known to lack an effective immune response with low immunogenicity, which results in rapid cancer progression and a limited response to cancer immunotherapy [7,8]. Despite the aggressive molecular behavior driven by intrinsic oncogenic genetic alterations, the tumor microenvironment (TME) of pancreatic cancer has been deemed to be responsible for the above dilemma [9,10,11,12].

PDAC is characterized by extensive deposition of desmoplastic stroma, which may comprise more than 80% of the whole tumor mass [13,14]. The extracellular matrix, vessels, and stromal cells comprise the TME of pancreatic cancer [15]. The cell component surrounding PDAC cells consists predominantly of cancer-associated fibroblasts, various immune cells, and endothelial cells. The complex interactions between TME cells and cancer cells contribute to tumor progression in a multifaceted way [16]. For example, cancer-associated fibroblasts, infiltrated inflammatory cells, and desmoplastic stroma enhance cancer growth, invasion, metastasis, and treatment resistance in direct or indirect ways [15,17]. The immune-suppressor cells in TME establish an immunosuppressive tumor microenvironment, which results in rapid cancer progression and a low immune response to immunotherapy [18]. Accumulating studies have revealed that the TME context correlates with clinical outcomes, therapy benefits, and immune response [19,20,21,22,23,24,25].

By now, although clinical decision-making based on molecular subtypes has been well established in some cancer types, subtypes of PDAC do not currently provide effective support for clinical decisions [26]. However, accumulating molecular subtypes have been defined in PDAC with the development of the genome project, which defined various PDAC subtypes with distinct tumor biological behaviors and clinical characteristics [27]. Although the several mechanisms associated with the role of TME have been highlighted in some previous subtypes [27,28,29,30,31], the comprehensive landscape of cells infiltrating the TME of PDAC has not yet been elucidated, as well as that there is a lack of a molecular subtype based on TME signatures to inform treatment decisions, including the applicability of immunotherapy. Therefore, in the present study, we evaluated the cell components of the TME in PDAC using computational algorithms and then established subtypes based on TME infiltration signatures. Finally, a robust TMEscore and an effective biomarker capable of risk stratification and informing treatment decisions were developed.

## 2. Materials and Methods

### 2.1. Sample Data Collection

The expression data (RPKM) of 182 PDAC patients were downloaded from The Cancer Genome Atlas (TCGA) data portal (https://portal.gdc.cancer.gov/ (accessed on 1 January 2020)) by using TCGAbiolinks. The fragments per kilobase of exon model per million (FPKM) data from TCGA were transformed into transcripts per kilobase per million (TPM) values. Additionally, other public PDAC datasets were obtained through the retrieval of the GEO database (https://www.ncbi.nlm.nih.gov/geo/ (accessed on 1 January 2020)) with the following retrieval strategy: (“pancreatic neoplasms” [MeSH Terms] OR pancreatic cancer [All Fields]) AND “Homo sapiens” [porgn] AND (“Expression profiling by array” [Filter]). Samples with survival data were retained for further analysis. The detailed information on the retrieved datasets was summarized in Supplementary Table S1.

### 2.2. Estimation of Cell Components in TME

The ESTIMATE algorithm was used to calculate the level of stromal cell and immune cell infiltration in each sample and further infer tumor purity [32]. Single-sample gene set enrichment analysis (ssGSEA) [33,34], a deconvolution algorithm based on gene set enrichment analysis (GSEA), was used to qualify the relative abundance of 29 cell types within the TME. The ssGSEA was run using the GSVA R package. Signature genes of each cell type were obtained from previous publications [35,36]. The ssGSEA score was normalized to a unity distribution, in which zero is the minimum score and one is the maximal score for each cell type. In some analyses, the immune infiltrations were also quantified by the GSVA algorithm [37], for which the normalized GSVA scores were obtained from a recent publication [38].

### 2.3. Consensus Clustering for Infiltrating Cells of TME

Unsupervised consensus clustering was performed on normalized ssGSEA scores of TME cell components by using the ConsensusClusterPlus R package (parameters: reps = 1000, pItem = 0.8, pFeature = 1). The complete method and Manhattan distance were used as the clustering algorithm and distance metric, respectively.

### 2.4. Identification of TME Signature Genes

To identify the signature genes of TME, we first estimated the differentially expressed genes (DEGs) associated with TME subtypes determined by consensus clustering of TME infiltration. The DEGs among TME subtypes were obtained using the limma R package with the selection criteria of Log2FoldChange > 1 and an adjusted *p*-value < 0.05 (Benjamini–Hochberg correction). Next, the random forest method was used to evaluate the contribution of these DEGs to the cluster grouping of the TME cell population, and the genes that had less influence on the grouping were filtered out. Finally, 74 DEGs that influenced the prognosis were obtained. An unsupervised clustering method (K-means) with Ward.D and Euclidean distance was used to classify patients into subgroups based on the 74 DEGs. The ConsensusClusterPlus R package was adopted and used to annotate gene patterns and define gene clusters.

### 2.5. Development of TMEscore

After obtaining the two gene clusters in the above part, the genes in each cluster were extracted to serve as the TME gene signature sets, respectively: TME signature gene set A from cluster 1, and TME signature gene set B from cluster 2. The ssGSEA algorithm was used to calculate the enrichment score of each TME signature gene set for each sample. Thereafter, the TMEscore for each sample was obtained by using the following formula: TMEscore = TMEscoreB − TMEscoreA, where TMEscoreB stands for the ssGSEA score of TME signature gene set B, TMEscoreA stands for the ssGSEA score of TME signature gene set A. A schematic diagram illustrating the process of generation of the TMEscore was described in Supplementary Figure S1.

### 2.6. Functional Annotation and Enrichment Analysis

A functional annotation analysis was conducted based on the GO database (http://geneontology.org/page/go-database (accessed on 1 January 2020)) and the KEGG database (http://www.kegg.jp/kegg/ko.html (accessed on 1 January 2020)). KEGG and GO term gene set enrichment analysis (GSEA) was conducted using the clusterProfiler R package. We also estimated the enrichment of pathways among TME gene clusters or samples with different TMEscores by running gene set enrichment analysis (GSEA) [33,39]. For GSEA analysis, gene sets for certain pathways were collected via searching the MSigDB database (https://www.gsea-msigdb.org/gsea/msigdb/ (accessed on 1 January 2020)). GSEA was performed using the GSEA software and visualized by the ggplot R package.

### 2.7. Mutation Analysis

TCGA-PAAD mutation data were downloaded in January 2020 from the GDC data portal. The copy number events were filtered for those with at least 10 supporting probes and a segment mean >0.2 (amplifications) or <−0.2 (deletions), as recommended by a previous study [40,41]. The waterfall plots of mutational landscapes were drawn using the maftools Bioconductor package [42]. Mutation types were ordered by their potential impact, from most deleterious to least.

### 2.8. Cell Lines and Cell Culture

Human cell lines BxPC-3, PANC-1, AsPC-1, Capan-2, MIA-PaCa2, SW1990, and hTERT-HPNE were purchased from the ATCC (American Type Culture Collection, Rockville, MD, USA). Cells were cultured in DMEM (Gibco, Billings, MT, USA) or RPMI 1640 medium (Gibco, USA). All media were supplemented with 10% fetal bovine serum (FBS, BI, Israel) and 1% penicillin/streptomycin. All cells were cultured in a humid environment containing 5% CO_2_ at 37 °C.

### 2.9. Cell Transfection and Lentiviral Infection

For cell transfection, the siRNAs for knocking down F2R-like Trypsin Receptor1 (F2RL1) and the F2RL1 overexpression plasmid were purchased from IGE (Guangzhou, China). Transfection was performed using the Lipofectamine 3000 kit (Invitrogen, Cat# L3000015, Waltham, MA, USA) according to the manufacturer’s instructions.

For lentivirus infection, in order to construct stable knockdown cell lines, the shRNA sequence of F2RL1 was cloned into a pLKO.1-Puro vector by IGE, and then the lentivirus packaging plasmids containing psPAX2 and pMD2G were cotransfected into HEK-293T cells (ATCC, RRID: CVCL_0063). After transfection for 72 h, lentivirus was collected and concentrated. Subsequently, the cell lines were infected with lentivirus and selected by treatment with puromycin (Solarbio, Beijing, China) for 2 weeks. All the sequences of oligonucleotides are shown in Supplementary Table S9.

### 2.10. RNA Extraction and qRT-PCR Analysis

The total RNA of PDAC cell lines was extracted using Trizol reagent (Takara Bio, Shiga, Japan) according to the instructions. Subsequently, the total RNA was reverse transcribed into cDNA using the Hiscript III Reverse Transcriptase Kit (Vazyme, Nanjing, China). Finally, qRT-PCR was used to detect the expression of RNA using the ChamQ Universal SYBR qPCR Master Mix kit (Vazyme, Nanjing, China). GAPDH was used as an internal control. The sequences of primers are listed in Supplementary Table S9.

### 2.11. Colony Formation Assay

A total of 500 cells transfected with siRNA or stably overexpressing F2RL1 were cultured in 6-well plates in a humidified atmosphere containing 5% CO_2_ at 37 °C for 2 weeks. After that, the colonies were fixed in 4% paraformaldehyde for 20 min, stained with 0.1% crystal violet for 15 min, and washed twice with phosphate-buffered saline (PBS). Count colonies manually. Each group repeated the experiment at least three different times.

### 2.12. EdU Assay

The cells transfected with siRNA or stably overexpressed F2RL1 were pre-seeded in 24-well plates and cultured at 37 °C containing 5% CO_2_ for 24 h. Then, using BeyoClickTM EdU-555 detection kits (Beyotime, Shanghai, China) and according to the manufacturer’s instructions, the cells were stained with EdU for 2 h, fixed with 4% paraformaldehyde for 15 min, incubated with click reaction solution for 30 min and stained with Hoechst 33,342 for 10 min. The images were obtained by fluorescence microscope Nikon TI-S (Nikon, Tokyo, Japan). Each group repeated the experiment at least three different times.

### 2.13. Wound Healing Assay

The cells transfected with siRNA or stably overexpressed F2RL1 were seeded in a 12-well plate according to 2 × 10^5^ cells/well and cultured in a moist environment containing 5% CO_2_ at 37 °C until the cell density was about 90%. Then adherent cells were scraped with the 10 μL sterile pipette tips in a straight line, and the images were obtained by an inverted microscope, the Nikon TI-S (Nikon, Tokyo, Japan), at 0 h and 24 h, respectively. The cell migration distance was measured and calculated. Each group repeated the experiment at least three different times.

### 2.14. Transwell Assay

The cells transfected with siRNA or stably overexpressed F2RL1 were added to 200 μL serum-free medium with or without Matrigel (BD Biosciences, Franklin Lakes, NJ, USA), respectively, and seeded in a Transwell chamber, then placed in a 24-well plate. 700 μL complete medium was added to each well in the lower layer of the 24-well plate in advance. PANC1 was incubated for about 8 h, and BxPC-3 was incubated for about 48 h. Then the Transwell chamber was removed, fixed with 4% paraformaldehyde for 15 min, and stained with 0.1% crystal purple for 15 min. Images were obtained by an inverted microscope, the Nikon TI-S (Nikon, Tokyo, Japan), and the number of cells that migrated or invaded was counted. Each group repeated the experiment at least three different times.

### 2.15. Animal Experiment

To construct a subcutaneous tumorigenicity animal model, 5 × 10^6^ BxPC-3 cells in suspension, stably overexpressing F2RL1 and Vector, were injected subcutaneously into the left dorsal side of BALB/c nude mice (*n* = 5) aged 4 to 5 weeks, purchased from the Guangdong Medical Laboratory Animal Center. Tumor growth was measured every 4 days, and tumor volume was recorded. Volume = 0.5 × length × width^2^. Four weeks later, all the mice were sacrificed, and the tumor tissue was dissected, weighed, and fixed with 37% formalin and embedded in paraffin.

### 2.16. Immunohistochemistry (IHC)

Immunohistochemical staining was performed on paraffin sections, which were first treated at 60 °C for 2 h, then dewaxed with xylene, rehydrated with different grades of ethanol, repaired antigen with EDTA, and blocked with normal goat serum. The sections were incubated with primary antibodies at 4℃ overnight and secondary antibodies at room temperature for 2 h. Finally, DAB chromogenic reagent was used to label the antigen, followed by counterstaining with hematoxylin. The staining was judged by two independent observers. Images were obtained by a microscope, the Nikon 80i (Nikon, Tokyo, Japan). The antibodies used in this study are listed in Supplementary Table S10.

### 2.17. Statistical Analysis

The distribution of two sets of continuous variables was compared using a *t*-test. If continuous variables did not follow a normal distribution, the Mann–Whitney U test was applied. Unless explicitly stated, the association between categorical variables was evaluated using Pearson’s chi-square test. To divide the samples assessed into groups according to high versus low TMEscore, the MaxStat R package was used. Survival curves were compared using the Kaplan–Meier method with a log-rank *t*-test. The influence of the TMEscore on survival was additionally evaluated through the Cox proportional hazard model. The independence of association was verified by a multivariate Cox regression model of survival. The resulting *p*-values of differently expressed genes between two groups were corrected for multiple testing by the Benjamini–Hochberg method. All reported *p*-values are two-sided. R (version 3.6.3) and SPSS (version 17.0; SPSS Inc., Chicago, IL, USA) were used to perform statistical analyses. Figures were generated with the ggplot R package and GraphPad Prism 8 (GraphPad Prism Software, San Diego, CA, USA). Two-sided *p* < 0.05 was considered significant.

## 3. Results

### 3.1. Identification of Tumor Microenvironment Subtypes in PDAC Cases

**Figure 1:**
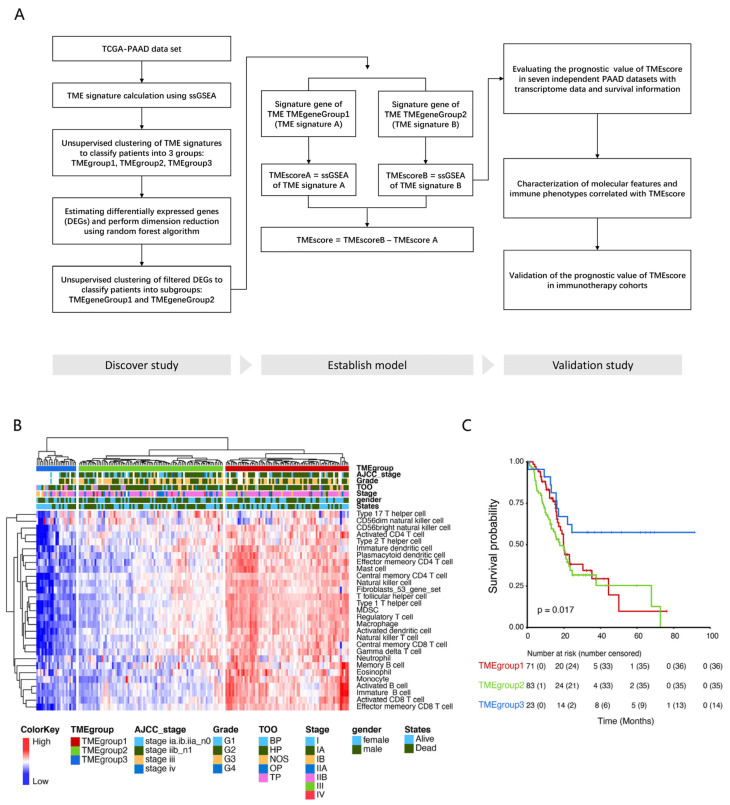
Identification of tumor microenvironment subtypes in PDAC cases. (**A**) Flow chart of the study. (**B**) We defined the infiltration pattern of a tumor as the relative abundance of TME cell populations estimated via ssGSEA. Unsupervised clustering of TME cells for the 177 samples in the TCGA-PAAD cohort was performed based on ssGSEA results. (**C**) Kaplan–Meier curves with a log-rank test for overall survival (OS) of 177 TCGA-PAAD patients stratified by TMEgroups.

The flow chart of the study is shown in Figure 1A. First, we defined the tumor microenvironment (TME) infiltration pattern of each tumor as the relative abundance of an array of twenty-eight cell populations of immune cells and fibroblasts. TME cell profiles were estimated via the ssGSEA algorithm (Supplementary Table S2). To select the optimal cluster number, we grouped the ssGSEA scores of the resectable PDAC tumors from the TCGA dataset using hierarchical clustering. As a result, we obtained three robust subtypes of PDAC (named TMEgroups1–3) (Figure 1B) (Supplementary Table S3). The TMEgroup3 is defined as the smallest group of cases (23/177, 13.0%), followed by TMEgroup1 (71/177, 40.1%), and TMEgroup2 (83/177, 46.9%). TMEgroup3 was associated with better overall survival in comparison with the other two TMEgroups (Log-rank test, *p* = 0.017, Figure 1C). In many solid tumors, the degree of immune infiltration of the TME is highly correlated with immunotherapy efficacy [43]. Due to the high tumor heterogeneity and TME heterogeneity in PDAC, the specific biological characteristics are different from other solid tumors [44]. As previous studies have shown, the abundance of immune cells tends to predict a poor prognosis [45,46]. The overall survival rate of TMEgroup3, which had the fewest immune cells, was higher than that of the other two groups.

### 3.2. Identification of the TME Signature Genes and Functional Annotation

**Figure 2:**
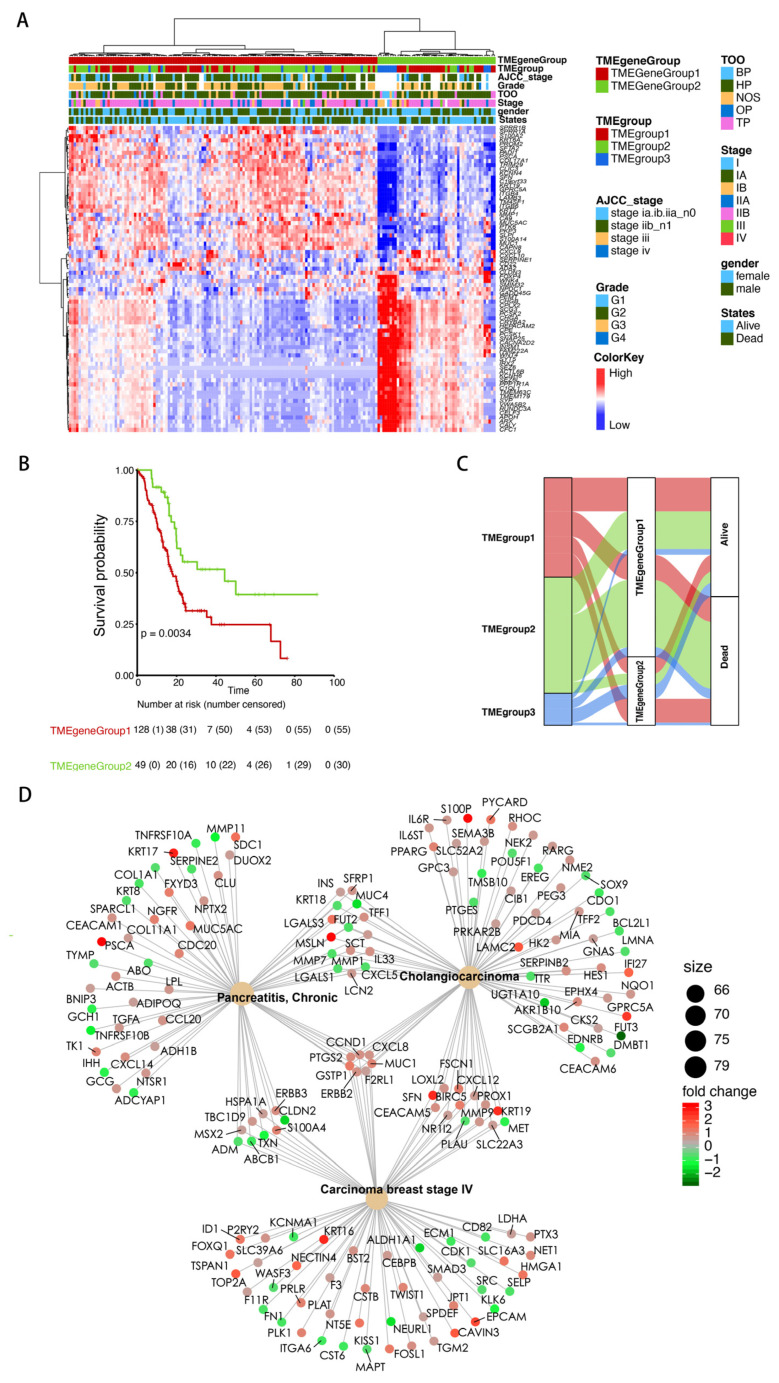
Identification of the TME signature genes and functional annotation. (**A**) Unsupervised analysis and hierarchical clustering of TME signature genes based on expression data derived from the TCGA-PAAD cohort were used to classify patients into two groups termed TMEgeneGroup1 and TMEgeneGroup2. (**B**) Comparison of the survival curves between the TMEgeneGroup1 and TMEgeneGroup2. (**C**) Alluvial diagram of TMEgroups with different TMEgeneGroups and survival outcomes. (**D**) Visualization of the potential regulatory network of TME signature genes revealed a close relationship with tumor progression.

To identify the signature genes associated with TMEgroups, differentially expressed genes (DEGs) between each TMEgroup and others were obtained using the limma R package, and the results are shown in Supplementary Figure S2 and Supplementary Table S4. As shown in the Venn diagram of Figure S2, there was no overlap between the DEGs from each TMEgroup, suggesting the high specificity of DEGs for each TMEgroup. Next, a random forest method was then used to estimate the contribution of these DEGs to the clustering of the TME cell population, and 74 genes with influence on the clustering were finally retained. By performing unsupervised hierarchical cluster analysis based on the 74 TME-related DEGs, we identified 2 robust groups for TCGA-PAAD samples: TMEgeneGroup1 and TMEgeneGroup2. The gene symbols for signature genes for each group were summarized in Supplementary Table S5. The patient-level annotation of the DEGs is visualized in Figure 2A. A significant decreased overall survival was found in TMEgeneGroup1 (Log-rank test, *p* = 0.0034, Figure 2B). The differences in class assignments between the two clustering methods were visualized with an alluvial diagram (Figure 2C). Most deaths occurred in TMEgroup3 and all deaths that occurred in TMEgroup2 were assigned to TMEgeneGroup1, suggesting signature genes had better prognostic discrimination values, such as the identification of patients at a high risk of death from subgroups with better prognosis.

In addition, we analyzed the tumor purity of the TCGA dataset, and the results showed that there was a difference between TMEgeneGroup1 and TMEgeneGroup2 in the tumor purity, suggesting that the tumor may be related to immune infiltration of TME (Supplementary Figure S2G, Supplementary Table S7). Further, we found that tumor purity was negatively correlated with the immune ssGSEA (Supplementary Figure S2H). Moreover, we further analyzed the laser microdissected dataset from Maurer C et al. [47]. Through the comparison of stromal and epithelial cells, we obtained 6308 DEGs representing stromal regions, which were further intersected with our 74 DEGs. Interestingly, the results showed that there were 41 duplicate DEGs (Supplementary Figure S2E), suggesting that those 41 DEGs we analyzed had the characteristics of representing stromal cells in non-tumor regions. Subsequently, we also verified the effect of ssGSEA used in this project in reflecting TME characteristics (Supplementary Figure S2F). The above results show that the difference in TME can be better reflected by screening effective DEGs to distinguish cell components.

To further explore the biological function and mechanism behind the signature genes, DEGs between TMEgeneGroup1 and TMEgeneGroup2 were determined (Supplementary Table S6). Functional annotation analysis of these DEGs was conducted. Significantly enriched KEGG pathways and GO biological processes were summarized in Supplementary Figure S3. We found TMEgeneGroup1 and TMEgeneGroup2 had distinct differences in the enriched pathways and biological processes. Genes overexpressed in TMEgeneGroup1 were involved in several well-known carcinogenesis mechanisms, such as the PI3K/Akt signaling pathway and the p53 signaling pathway. In addition, mechanisms involved in extracellular matrix composition, cell-extracellular matrix interaction, and cell adhesion were enriched in TMEgeneGroup1. In contrast, genes overexpressed in TMEgeneGroup2 were mainly involved in signal molecule transduction-related mechanisms, such as the cAMP signaling pathway, ligand-receptor interaction, single release, chemical synaptic transmission, and regulation of membrane potential. Furthermore, using disease network enrichment analysis with all DEGs between TMEgeneGroup1 and TMEgeneGroup2 (Figure 2D), we found enriched disease gene sets related to chronic pancreatitis, cholangiocarcinoma, and breast carcinoma stage IV. In addition, we identified seven core DEGs: GSTP1, ERBB2, MUC1, F2RL1, PTGS2, CCND1, and CXCL8, which were involved in all three gene sets.

### 3.3. Establishment of TMEscore Model Based on TME Signature Gene Sets

As mentioned above, the unsupervised hierarchical cluster analysis was based on the 74 most representative DEGs and separated the PDAC cohort into 2 distant patient clusters (Figure 2A). Subsequently, the 74 DEGs were divided into 2 distinct clusters, termed TME signature gene set A (enriched in TMEgeneGroup1) and TME signature gene set B (enriched in TMEgeneGroup2). Furthermore, based on the two TME signature gene sets, we estimated two TME-related scores using the ssGSEA algorithm as described in the “Methods” part: TMEscoreA from TME signature gene set A and TMEscoreB from TME signature gene set B, thus obtaining the final TMEscore through the following formula: TMEscore = TMEscoreB − TMEscoreA.

**Figure 3:**
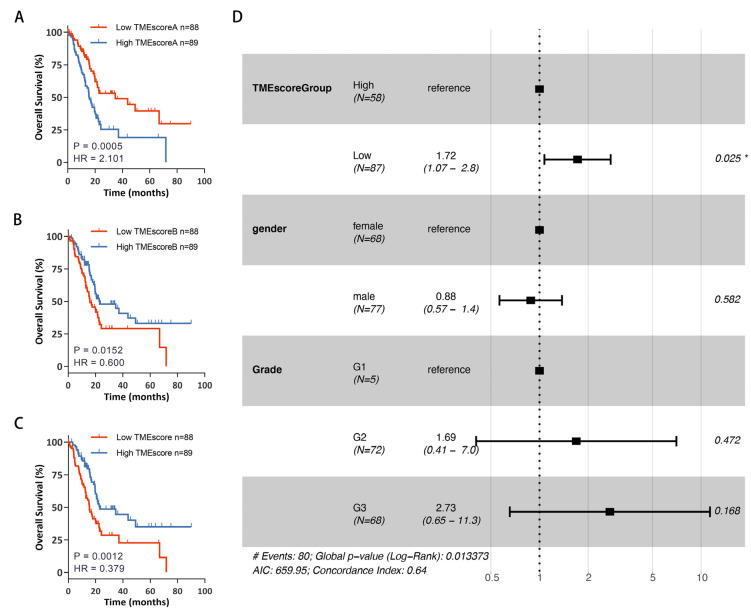
Prognostic value of the TMEscore. (**A**) Kaplan–Meier curves for high and low TMEscoreA patient groups in the 177 TCGA-PAAD patients. (**B**) Kaplan–Meier curves for high and low TMEscoreB patient groups in the 177 TCGA-PAAD patients. (**C**) Kaplan–Meier curves for high and low TMEscore patient groups in the 177 TCGA-PAAD patients. (**D**) Univariate and multivariate analyses were conducted to evaluate the prognostic value of TMEscore and histologic prognostic factors. Hazard ratios (HR) > 1.0 indicate that a low TMEscore is an unfavorable prognostic biomarker. (*: *p* < 0.05).

After having identified the TMEscore for each patient in the TCGA-PAAD cohort, we sought to determine whether the TMEscore could effectively predict prognosis. As shown in Figure 3A–C, low TMEscoreA was correlated with improved survival (Log-rank test, *p* = 0.0005) in TCGA-PAAD patients, and a low TMEscoreB was associated with poor survival (Log-rank test, *p* = 0.00152). At last, survival analysis revealed that patients with a low TMEscore had a less favorable outcome (Log-rank test, *p* = 0.00065). Multivariate Cox models revealed that the TMEscore was an independent prognostic variable for overall survival (HR = 1.72. 95%CI 1.07–2.80, *p* = 0.025) (Figure 3D). Next, we tried to validate the prognostic value of TMEscore with seven external data sets obtained from the GEO database. As shown in Supplementary Figure S4, upon stratification of the samples according to TMEscore, significant differences in overall survival were found between the TMEscore low and high groups for all datasets except GSE28735 (*p* = 0.14, sample size = 43, the dataset with the lowest sample size), which confirmed the robust prognosis stratification ability of the TMEscore.

### 3.4. Exploring the Biological Characteristics of Patients with Different TMEscore

**Figure 4:**
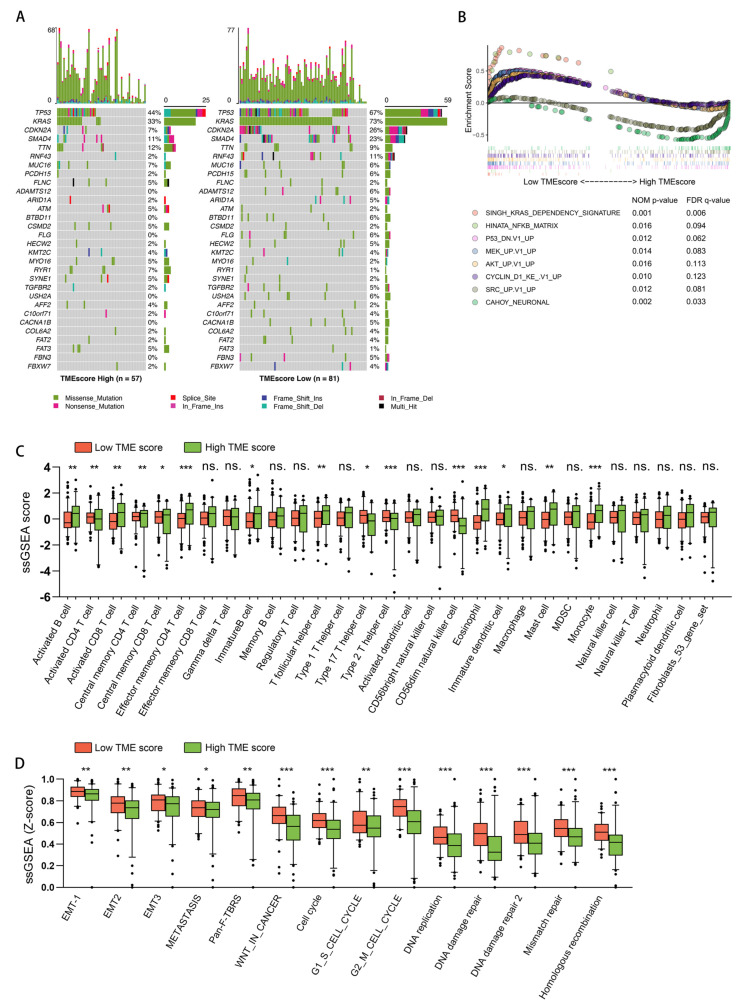
Relationship between the TMEscore and biological characteristics and TME features of pancreatic cancer. (**A**) A waterfall plot for the distribution of highly variant mutated genes with TMEscore. (**B**) Gene set enrichment analysis (GSEA) of two gene sets: the KEGG gene sets (c2.cp.kegg.v6.2.symbols) and oncogenic signature gene sets (c6.all.v6.2.symbols). Samples were ranked by TMEscore. (**C**) TME cell infiltrations in the TMEscore-high and TMEscore-low patients were compared. (**D**) Gene signatures describing multiple pathways correlated with immune activity were quantified by ssGSEA. The normalized ssGSEA scores between TMEscore-high and TMEscore-low patients were compared. Box plots show the differences in the enrichment of each gene set between TMEscore high and low patients. Each dot represents the z-score normalized ssGSEA result. The thick line of the box plot represents the median value. The bottom and top of the box indicate the upper and lower quartiles, respectively. (ns.: not significant, *: *p* < 0.05, **: *p* < 0.01, ***: *p* < 0.001).

To explore the underlying molecular mechanisms associated with the TMEscore, we first compared the profile of oncogenic mutations between patients with a low and high TMEscore (Figure 4A). We found significantly increased mutation rates in KRAS, TP53, and CDKN2A in the low TMEscore group. Afterward, the GSEA was performed to evaluate the pathways associated with the TMEscore (Figure 4B). The significantly enriched gene sets in samples with a low TMEscore were correlated with KRAS, NF-κβ, P53, MEK, AKT, and cell cycle signaling pathways, all directly associated with tumor development. On the other hand, genes up regulated in neurons and in response to overexpressing Src were enriched in samples with a high TMEscore. The GSEA results were consistent with the differences in mutation spectras between patients with a low and high TMEscore. Additionally, these results also indicated that the TME-based score could reflect tumor-intrinsic mechanisms at the level of driver gene profiles.

Next, we analyzed the infiltration of cell populations between two groups. Figure 4C displays the differences in TME cell infiltration in the two groups with high and low TMEscores. Overall, most cell populations were increased in samples with a high TMEscore, especially since the infiltration of activated CD4/CD8 T cells was significantly increased. In light of the well-recognized close relationship between TME and immune status, we further compared the well-known biological mechanisms/pathways associated with TME and cancer-immune phenotypes between the two groups. The feature genes for each mechanism/pathway were summarized in Supplementary Table S8. As shown in Figure 4D, the ssGSEA analysis confirmed a significant enrichment of genes representing EMT, the TGF-β pathway, the Wnt pathway, homologous recombination, mismatch repair, and DNA damage repair in low TMEscore samples. This suggests that the TMEscore may reflect tumor environment infiltrations, tumor-intrinsic mechanisms, and immune status.

### 3.5. Characterization of Immune-Phenotypes across Samples with Different TMEscore

**Figure 5:**
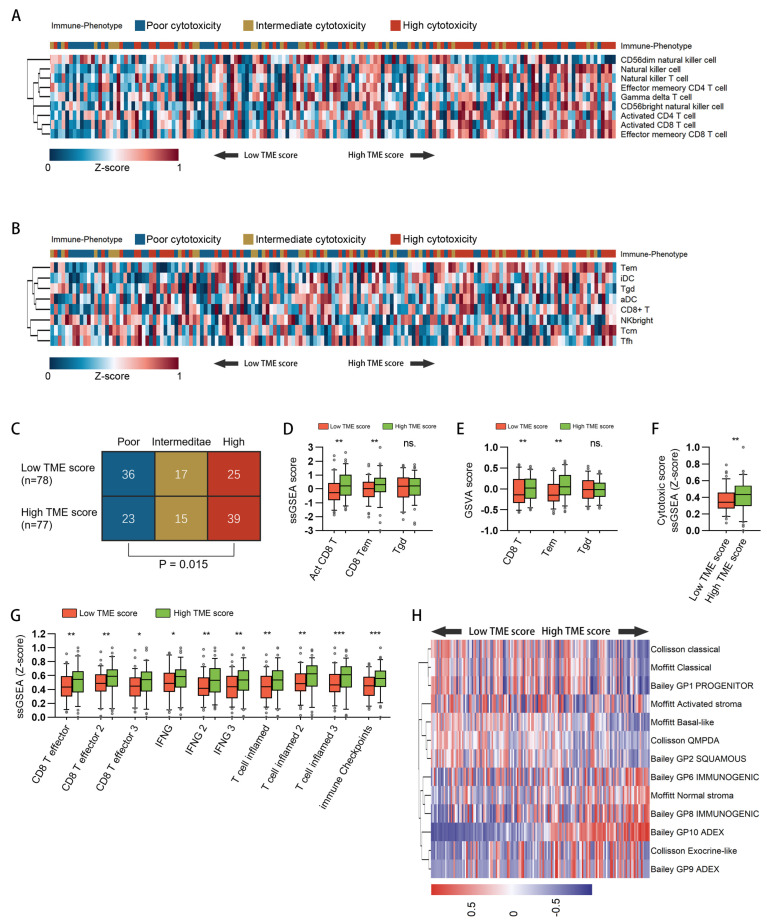
Association between immunophenotypes and the TMEscore. (**A**) A heatmap showing the association between well-established immune phenotypes, ssGSEA results of cytotoxic cells, and TMEscore in 156 TCGA-PAAD samples. Samples were ranked by TMEscore. The immune phenotypes and z-score normalized ssGSEA results are shown as patient annotations. (**B**) Cell infiltration was further estimated by gene set variation analysis (GSVA). The samples were ranked by TMEscore, and the immune phenotypes and z-score normalized GSVA results are shown as patient annotations. (**C**) Chi-square test of the difference in TMEscore between groups of different immune phenotypes. (**D**,**E**) The infiltration of main cytotoxic cells was compared by either ssGSEA (**E**) or GSVA (**E**). (**F**) Cytotoxic scores between TMEscore high and low patients were compared. (**G**) Gene sets of the T-cell inflamed phenotype were evaluated by ssGSEA. Box plots show the differences in the enrichment of each gene set between TMEscore high and low patients. (**H**) Hierarchical clustering of GSVA signature scores for gene programs defining PDAC subtypes. (ns.: not significant, *: *p* < 0.05, **: *p* < 0.01, ***: *p* < 0.001).

To further delineate the link between TMEscore and immune status, we evaluated the relationship between a well-established immune phenotype and TMEscore. The immune phenotypes of 156 TCGA-PAAD samples were obtained from a previous publication, which classified tumors into three immune phenotypes: poor cytotoxicity, intermediate cytotoxicity, and high cytotoxicity on the basis of cytotoxic infiltration [38]. The abundance of cytotoxic cells was estimated by either ssGSEA (Figure 5A) or GSVA (Figure 5B). As shown in the heatmaps (Figure 5A,B) and the result of the chi-square test (Figure 5C), we observed that the cytotoxic level of the immune phenotype tended to increase with the elevated level of TMEscore. A previous study reported that tumors with a highly cytotoxic immune phenotype tend to show an increased abundance of cytotoxic infiltration with ectopic expression of negative immune checkpoints [38]. So, we next estimated the infraction of activated CD8 T cells, effector memory T cells, and γδ T cells by ssGSEA (Figure 5D) and GSVA (Figure 5E). Both algorithms showed increased infiltration of CD8 T cells and effector memory T cells in samples with high TMEscores. Additionally, we observed the same increase in the cytotoxic score (Figure 5F). These results collectively suggested that a high TMEscore indicates a TME with high cytotoxic activity. Therefore, we next estimated the enrichment of gene sets associated with the T cell–inflamed phenotype, which correlates with improved responsiveness to therapies dependent on T cell killing, such as checkpoint blockade and adoptive cell therapy [48]. We used three independent gene sets for each comparison, including gene sets that have been confirmed to be predictive of response to immunotherapy across different cancer types. Notably, all gene sets displayed significant enrichment in the high TMEscore group (Figure 5G). Meanwhile, we also observed the enrichment of immune checkpoints.

At last, we assessed the enrichment of gene programs defining PDAC subtypes and their association with the TMEscore (Figure 5H). The expression of genes defining the ADEX (aberrantly differentiated endocrine exocrine) subtype [30] was increased in TMEscore-high tumors, while the genes defining the pancreatic progenitor subtype tended to increase in TMEscore-low tumors. TMEscore-high tumors were statistically enriched for the immune gene programs (GP6 and GP8) from Bailey and colleagues [30]. These two gene programs contain signature genes for CD8+ T cells and B cells, supporting the finding that TMEscore-high tumors had high cytotoxic infiltration. Furthermore, TMEscore-high tumors were enriched for the normal stroma gene program. The normal stroma gene signature contains markers of pancreatic stellate cells, which have been linked to an immunosuppressive tumor microenvironment through blocking antigen presentation [29,49,50]. In contrast, TMEscore-low tumors were enriched for the activated stroma gene program. The activated stroma was characterized by a more diverse set of genes associated with activated fibroblasts and activated inflammatory stromal responses, both of which were responsible for a low antitumor immune response [29].

### 3.6. The Predictive Value of TMEscore for Response to Immune Therapy

**Figure 6:**
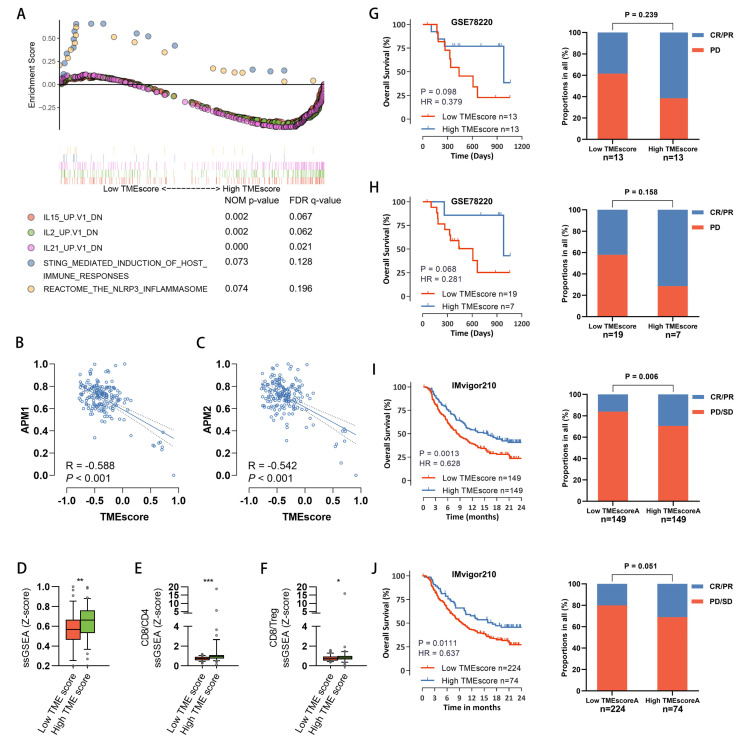
TMEscore as a prognostic marker for immunotherapy. (**A**) GSEA of immunologic signature gene sets downloaded from MSigDB database (c7.all.v6.2). Samples were ranked by TMEscore. (**B**,**C**) The antigen presentation activity was measured by ssGSEA based on two gene sets (APM1 and APM2; detailed information was supplied in Supplementary Table S8). The correlation between normalized ssGSEA scores and the TMEscore was evaluated by Pearson’s correlation analysis. (**D**) The abundance of Batf3-DC in each TCGA-PAAD sample was quantified by ssGSEA. The Batf3-DC scores between the TMEscore high and low groups were compared. (**E**,**F**) Ratios of CD8/CD4 and CD8/Treg in each sample were calculated using normalized ssGSEA results. Box plots show the differences in CD8/CD4 (**E**) and CD8/Treg (**F**) between TMEscore high and low groups. (**G**,**H**) Patients in the GSE78220 dataset were divided into two groups according to the median TMEscore (**G**) or upper interquartile TMEscore (**H**). Survival outcomes between patients with high and low TMEscores were compared by Kaplan–Meier curves with the log-rank test. Additionally, the ratios of response to immunotherapy were compared by Pearson’s chi-square test. (**I**,**J**) Patients of the IMvigor210 cohort were also divided into two groups based on TMEscore. The survival outcomes and responses to immunotherapy were compared. (*: *p* < 0.05, **: *p* < 0.01, ***: *p* < 0.001).

The above data suggested that the TMEscore can stratify PDAC patients into distinct clusters or subtypes not only with different tumor-intrinsic characteristics but also with different stromal statuses and immune environments. To further validate this finding, we performed GSEA and found enrichment of immune sensing pathways involved in T cell priming (STING and NLRP3 inflammasome signaling) in TMEscore-low tumors (Figure 6A). We found that both STING and NLRP3 inflammasome signaling were enriched in samples with low TMEscore. Consistent with this, the antigen presentation activity, which was measured by the ssGSEA score of two independent gene sets reflecting the antigen presentation mechanism (APM), was elevated in samples with a low TMEscore (Figure 6B,C). Interestingly, the expression of signature genes of Batf3-dendritic cells, a key antigen-presenting cell population for driving T cell immunity and response to immunotherapy in PDAC [51,52], was increased in TMEscore-high tumors (Figure 6D). These results support the above hypothesis that a low TMEscore identified tumors with normal stroma status, which was characterized by blocked antigen presentation. In addition, we found both the CD8/CD4 ratio and the CD8/Treg ratio were significantly increased in TMEscore-high tumors (Figure 6E,F). Both of them were biomarkers of elevated cytotoxic activity. We also found that downregulated genes after IL15, IL2, or IL21 stimulation were enriched in TMEscore-high tumors (Figure 6A). IL15, IL2, and IL21 were responsible for the expansion of cytotoxic T cells; therefore, the negative correlation between TMEscore and the expression of these gene sets supported a cytotoxic environment with exhaustion in TMEscore-high tumors. Collectively, the stratification of patients with TMEscore based on transcriptional profiling may differentiate between tumors with different immune evasion mechanisms and different immunotherapy responses.

Immune checkpoint blockade therapy, such as inhibitors targeting the PD1–PDL1 axis, shows promising prospects for cancer treatment. We subsequently explored the prognostic value of the TMEscore in patients who received immune-checkpoint therapy. As there is currently no available cohort with both transcriptome and survival information for immune therapy in pancreatic cancer, we used two well-known solid tumor cohorts receiving immune checkpoint blockade therapy. As shown in Figure 6G–J, patients with high TMEscores had significantly longer overall survival than those with low TMEscores in both the GSE78220 [53] cohort (anti-PD1) and IMvigor210 [54] cohort (anti-PDL1). In line with survival analysis, patients with a high TMEscore also showed an increased response rate to both anti-PD-1 (GSE78220) and anti-PD-L1 (IMvigor210) antibody treatment. Taken together, our data suggest that TMEscore could predict the response to checkpoint inhibitor immunotherapy.

### 3.7. F2RL1 Was Significantly Associated with the Malignant Progression of PDAC

**Figure 7:**
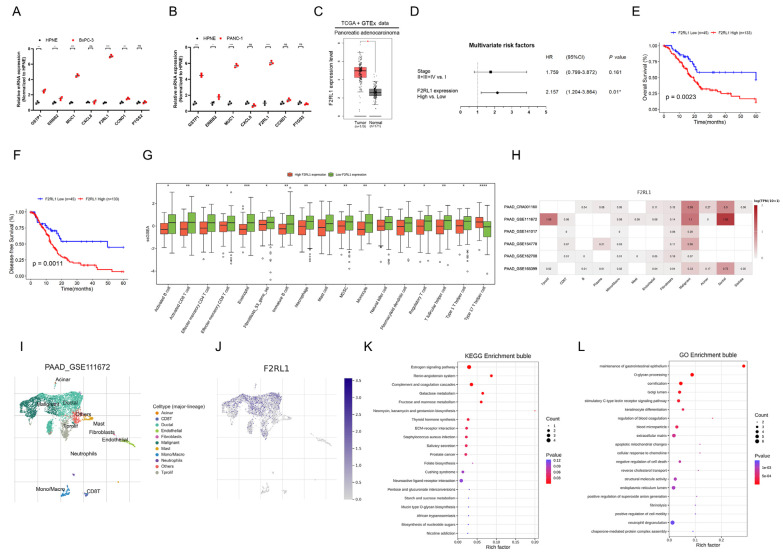
Upregulated F2RL1 is associated with malignant progression of PDAC. (**A**,**B**) qRT-PCR analysis of core DEGs expression between BxPC-3 and PANC-1 cell lines. (**C**) TCGA and GTEx database analysis showed the expression of F2RL1 in tumor and non-tumor tissues of PDAC. (**D**) Multivariate Cox analysis of the PDAC cohort in the TCGA database. (**E**,**F**) Kaplan–Meier survival analysis of OS (**E**) and DFS (**F**) in patients with low and high F2RL1 expression of PDAC. (**G**) The correlation analysis between the expression of F2RL1 and ssGSEA. (**H**) Heat maps showing the expression of F2RL1 in different annotated cells in single-cell PDAC data from the TISCH database. (**I**,**J**) Cell types and distribution in the GSE111672 dataset (**J**), and expression distribution of F2RL1 in different cells (**J**). (**K**,**L**) The KEGG pathways (**K**) and GO biological processes (**L**) enrichment analysis of F2RL1 in malignant cells. (ns.: not significant, *: *p* < 0.05, **: *p* < 0.01, ***: *p* < 0.001, ****: *p* < 0.0001).

To further explore potential drug therapeutic targets for PDAC, we screened the aforementioned core DEGs: GSTP1, ERBB2, MUC1, F2RL1, PTGS2, CCND1, and CXCL8, and verified the expression between BxPC-3 and PANC-1 cell lines. The results showed that the expression of F2RL1 was significantly high (Figure 7A,B). Currently, the mechanism of F2RL1 in the malignant progression of pancreatic cancer remains unclear, and more scientific evidence is needed. TCGA and Genotype-Tissue Expression (GTEx) database analysis showed that F2RL1 was highly expressed in PDAC tumor tissues compared with non-tumor tissues (NAT) (Figure 7C). Then, univariate and multivariate Cox analyses revealed that F2RL1 was an independent influence factor on the overall survival (OS) and disease-free survival (DFS) of PDAC patients (Figure 7D, Supplementary Tables S11 and S12). Importantly, Kaplan–Meier analysis showed that PDAC patients with high F2RL1 expression had shorter OS and DFS, suggesting that F2RL1 is associated with the malignant progression of PDAC (Figure 7E,F). Notably, further ssGSEA correlation analysis showed that the expression of F2RL1 was related to some immune cells in TME (Figure 7G). Further, we used the TISCH platform (http://tisch.comp-genomics.org (accessed on 2 January 2023)) to analyze the expression of F2RL1 at the cellular level. The results showed that F2RL1 was mainly distributed in malignant cell subsets (Figure 7H–J). Moreover, through functional annotation analysis of malignant cells, the enrichment results of the KEGG pathway and GO biological process showed that F2RL1 was mainly related to extracellular matrix composition, signaling pathways, cell adhesion, and other mechanisms (Figure 7K,L). The above results suggested that the upregulation of F2RL1 could promote the malignant progression of PDAC.

### 3.8. As an Effective Therapeutic Target, F2RL1 Promotes the Proliferation and Invasion of PDAC In Vitro and Vivo

**Figure 8:**
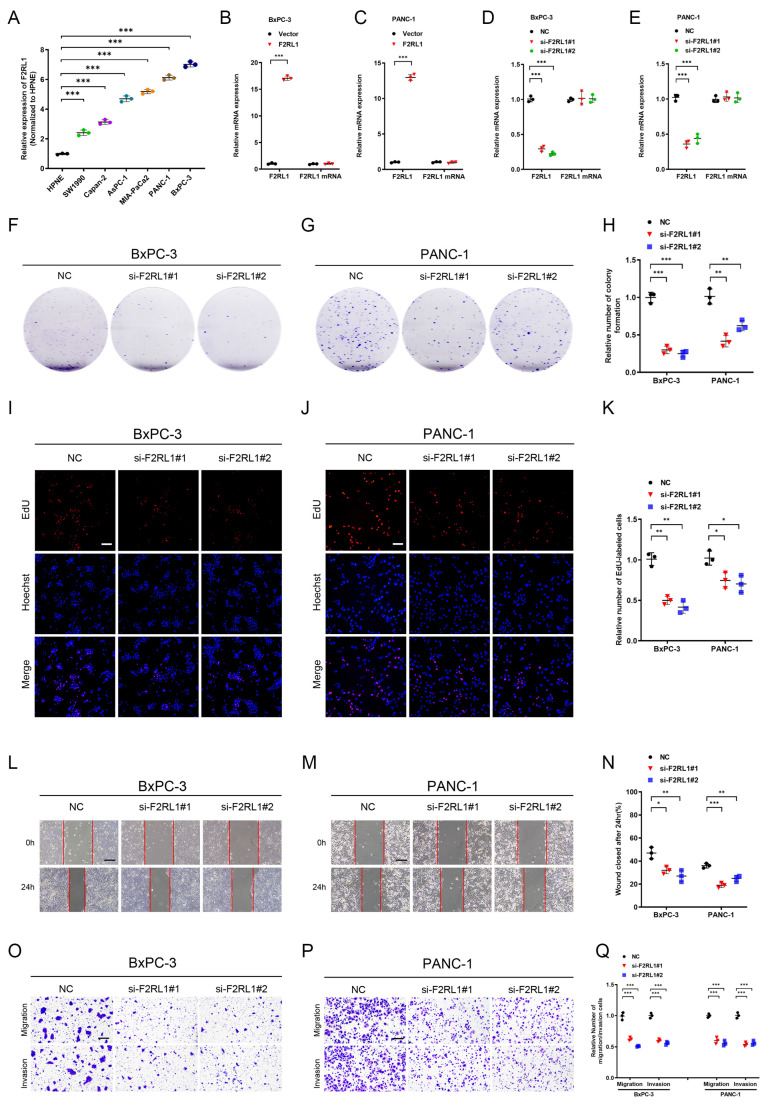
Knockdown F2RL1 inhibits proliferation, migration, and invasion of PDAC cells in vitro. (**A**) The expression of F2RL1 in normal pancreatic epithelial cells and PDAC cell lines was analyzed by qRT-PCR. (**B**–**E**) The qRT-PCR was used to analyze the silenced and overexpression of F2RL1 in BxPC-3 and PANC-1. (**F**–**H**) The colony formation assay showed representative images (**F**,**G**) and quantitative analysis (**H**) of BxPC-3 and PANC-1 after knocking down F2RL1. (**I**–**K**) EdU assay showing representative images (**I**,**J**) and quantitative analysis (**K**) of BxPC-3 and PANC-1 after knocking down F2RL1. Scale bars: 100 μm. (**L**–**N**) Wound healing assay showing representative images (**L**,**M**) and quantitative analysis (**N**) of BxPC-3 and PANC-1 after knocking down F2RL1. Scale bars: 100 μm. (**O**–**Q**) Transwell assay showing representative images (**O**,**P**) and quantitative analysis (**Q**) of BxPC-3 and PANC-1 after knocking down F2RL1. Scale bars: 100 μm. Error bars represent the standard deviations from three independent experiments. (*: *p* < 0.05, **: *p* < 0.01, ***: *p* < 0.001).

Given that F2RL1 was associated with a poor prognosis for PDAC, we further explored its biological function. We analyzed the expression of F2RL1 in PDAC cell lines, and the results showed that the expression of F2RL1 was significantly high in PANC-1 and BxPC-3 (Figure 8A). Further, we verified the transfection efficiency of F2RL1 in BxPC-3 and PANC-1 cell lines by knockdown and overexpression of F2RL1 (Figure 8B–E). The colony formation assay (Figure 8F–H, Supplementary Figure S5A–C) and EdU assay (Figure 8I–K, Supplementary Figure S5D–F) showed that the proliferation ability of cells, compared with the control group, was decreased after F2RL1 expression was down-regulated. The overexpression of F2RL1 showed the opposite effect. These results suggested that the upregulation of F2RL1 can promote the proliferation of PDAC cells. The transwell assay (Figure 8O–Q, Supplementary Figure S5J–L) and the wound healing assay (Figure 8L–N, Supplementary Figure S5G–I) showed that, compared with the control group, the cell invasion ability was weakened after F2RL1 was down-expressed, while the effect was opposite after F2RL1 was over-expressed. Therefore, these results indicated that the overexpression of F2RL1 could promote the proliferation and invasion of PDAC cells in vitro.

**Figure 9:**
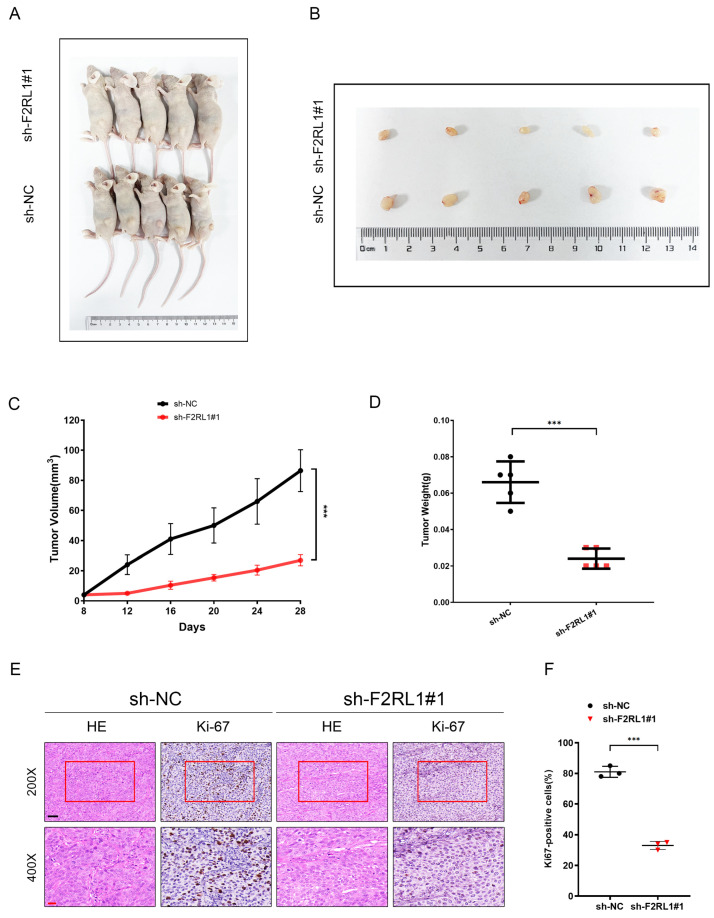
F2RL1 promotes PDAC tumorigenesis in vivo. (**A**,**B**) Representative images of subcutaneous xenograft tumors. (**C**,**D**) tumor volume (**C**) and weight (**D**) of the corresponding group. (**E**) Representative images of HE and IHC in subcutaneous tumor tissue. Scale bars: 50 μm (black) or 20 μm (red). (**F**) Histogram analysis showed a quantitative analysis of IHC staining for Ki67 expression. Error bars represent the standard deviations from three independent experiments. (***: *p* < 0.001).

Further, we verified the carcinogenic function of F2RL1 in a subcutaneous tumorigenicity mouse model (Figure 9A,B). Animal experiments showed that, compared with the sh-NC group (*n* = 5), the tumor volume and weight in the sh-F2RL1#1 group were lower (Figure 9C,D). Remarkably, IHC staining showed lower levels of Ki-67 expression in stable knockdown F2RL1 tissues (Figure 9E,F). Therefore, the downregulation of F2RL1 can strikingly inhibit the proliferation of PDAC in vivo.

Collectively, F2RL1 may be a potential biomarker for predicting survival outcomes in patients with PDAC.

## 4. Discussion

It is well acknowledged that the TME is of vital importance in cancer progression and therapeutic responses [55]. In this context, we evaluated the infiltration pattern of TME cells through the computational integration of their signature genes and developed a TMEscore that robustly predicts the prognosis for PDAC patients. Through comparison with the established PDAC molecular subtypes, we found the TMEscore differed across multiple established PDAC subtypes. Overall, tumors with a high TMEscore tend to share transcriptional commonalities with ADEX/Exocrine-like subtypes, which were defined by the transcriptional expression of multiple genes associated with terminally differentiated pancreatic tissues [30]. Instead, tumors with a low TMEscore were enriched with squamous/classical subtypes which reflect the molecular characteristics of squamous tumors across multiple tissue types, such as hypoxia response, metabolic reprogramming, and TGF-β signaling [30]. The close relationship between the TMEscore and tumor essential character-based subtypes suggested a correlation between TME and tumor cell-intrinsic properties. On the other hand, as expected, TMEscore-low tumors showed a subtype of “activated stroma” [29], while tumors with a high TMEscore displayed a “normal stroma” subtype [29]. Beyond that, TME high-score tumors were enriched for the immune gene programs (GP6 and GP8) from Bailey and colleagues [30]. These two gene programs were associated with B cells and CD8+ T cell infiltration signatures and T cell co-inhibitory phenotypes, respectively [30]. Therefore, the TMEscore is a comprehensive index reflecting tumor intrinsic features, stromal states, and immunophenotype.

PDAC is strikingly resistant to traditional treatment [56,57,58]. Immune checkpoint inhibitors, represented by PD-1/PD-L1 blockers, are widely believed to be a promising modality in pancreatic cancer, but the high prevalence of immunotherapy resistance of PDAC remains a main obstacle [59]. Effective identification in PDAC patients with potential benefits from immunotherapy could facilitate the translation of immunotherapy agents from preclinical research to clinical trials or applications. However, the biomarker for the efficiency of immunotherapy is thus far lacking [60]. A strong relationship between the tumor mutational burden (TMB) and the activity of immune checkpoint inhibitor (ICI) therapies has been identified across multiple cancers, but not pancreatic cancer [61]. MMR-D pancreatic cancer has been reported to respond to checkpoint inhibitor therapy, but it occurs in less than 1% of PDAC patients [62,63]. The MSI-H/dMMR phenotype is also very rare in PDAC [62]. The essential role of TME in immunosuppressants has been well known, and the impact of TME on the immune behavior of tumors has become a research focus in the immunotherapy of PDAC [9,64,65,66]. Therefore, personalized immunotherapy for solid tumors can be realized based on the characteristics of TME cell components. With the help of several computational algorithms, the TMEscore established in this study could represent the landscape of the whole infiltration. Notably, in the context of the lack of immunotherapy in the PDAC cohort, we also determined that a high TMEscore is associated with increased response to anti-PD-L1/anti-PD-1 agents and improved survival time in two cohorts of patients with other solid tumors. Therefore, our present TMEscore might help predict the response to immune checkpoint inhibitors and thus promote the precision immunotherapy of PDAC.

The predictive value of our TMEscore was supported by some facts. The finding that cytolytic activity in PDA did not correlate with TMB or neoantigen load reveals the distinct difference between pancreatic and others in antitumor immunity [50]. It seems that the immune privilege of pancreatic cancer depends much more on intrinsic oncogenic processes than that of other cancers [50]. Several intrinsic oncogenic mechanisms responsible for the immunosuppression in pancreatic cancer have been identified, including the Kras mutation [67,68], the CDKN2A mutation [69], the TGF-beta within the TME [70], the activation of WNT/beta-catenin signaling [71], PTEN loss, and PI3K–AKT pathway activation [72,73,74,75,76], which were all highly enriched in TMEscore-low tumors. The essential role of TME as an immunosuppressant has been well known, and the heterogeneous TME shaped by infiltrated cells might illustrate the underlying mechanisms of altered response to immunotherapy. Importantly, a low TMEscore may indicate the presence of stubborn intrinsic immunosuppression. Taken together, different TME cell components are reflected according to different immune infiltration subtypes, which has significant clinical value for further guiding the refinement of immunotherapy.

Despite the tumor-intrinsic acquired resistance, tumor-extrinsic acquired resistance also contributes to immunosuppression and resistance to immunotherapy [77,78]. The tumor-extrinsic immune microenvironment can be mostly reflected by the evaluation of the degree of T cell-inflamed phenotype. A baseline T cell-inflamed TME phenotype has been demonstrated to correlate with responsiveness to checkpoint blockade therapy and adoptive cell therapy [48]. Multiple T cell-inflamed gene signatures have been established and proven to be correlated with clinical response to PD-1PD-/L1 blockade across a variety of tumor types. Through assessing the T cell-inflamed status of PDAC tumors via the well-established signatures, including T cell-inflamed signatures [79], IFN-related genes [79], effector T cell signatures [80], and cytotoxic genes [81], we found significant enrichment of all signatures in TMEscore-high tumors. In addition, ratios of CD8+ to CD4 and Treg were also elevated in TMEscore-high tumors. All these results support the TMEscore-high tumors have T cell-inflamed phenotypes and vice versa for the TMEscore-low tumors. Despite the T cell-inflamed phenotype, there is other evidence providing support for a negative correlation between resistance to immunotherapy and TMEscore. It is known that NLRP3 signaling drives resistance to anti-PD-1 immunotherapy and is responsible for adaptive immune suppression through promoting the production of IL-1β in pancreatic carcinoma [82]. Our GSEA results indicated an underlying enrichment of NLRP3-related genes in TMEscore-low tumors. Furthermore, the gene sets associated with down-regulated genes in response to the stimulation of IL-2, IL-15, or IL-21 in T cells were negatively enriched. The impaired response to the proliferation stimulus suggested a dysfunctional state of infiltrated cytotoxic cells. The enrichment of multiple inhibitory checkpoint molecules in the group with a high TMEscore was also observed. Collectively, the TMEscore-high tumor has a series of characteristics suitable for immunotherapy.

There are some interesting findings that should be noted in our present study. F2R-like Trypsin Receptor 1 (F2RL1), as a G-protein-coupled receptor, can be activated by serine proteases and plays a key role in tumor progression [83]. We have confirmed that the upregulation of F2RL1 in PDAC tumors can significantly promote tumor proliferation, invasion, and migration. Moreover, it has been reported that the enrichment of F2RL1 in the tumor matrix region is increased [84,85], which is also similar to our results, suggesting that it mediates TME matrix remodeling to further drive tumor malignant progression. Therefore, whether there are subsets of cells with F2RL1 as a marker in the TME of PDAC as a bridge or not, the interaction between the tumor and TME remains to be further explored. The exact site structure of receptors and ligands also needs to be clarified. This provides new insights into individualized treatment schemes based on risk stratification based on the TMEscore and targeted therapy for F2RL1. In addition, the STING pathway, a cytosolic DNA sensing pathway, is a proximal event required for optimal type I interferon production, dendritic cell activation, and priming of CD8+ T cells against tumor-associated antigens [86]. STING pathway activation with antigen-presenting cells in the tumor microenvironment leads to the spontaneous generation of antitumor CD8+ T-cell responses [86]. Interestingly, we found the STING pathway was enriched in low TMEscore samples, which was somewhat at odds with that. Low TMEscore was associated with low cytotoxic infiltration and low T cell-inflamed activity, but the high APM score of TMEscore-low samples was in coherence with the enrichment of the STING pathway. The different STING activity and antigen presentation scores between TMEscore high and low samples could potentially be explained by the difference within TME. For example, the normal stromal phenotype was considered associated with the immunosuppressive pancreatic stellate cells, which inhibit the function of dendritic cells [29]. A previous study has reported that combining STING-based agonists with checkpoint modulators could enhance antitumor immunity in murine pancreatic cancer [87]. Therefore, considering the character of the low STING activity, high TME group, the combination of STING pathway agonists with immune checkpoint inhibitors seems to be a promising strategy in these patients.

Taken together, the present TMEscore was associated with a variety of molecular hallmarks of immunosuppression and antitumor immunity. The prognostic value of the TMEscore was validated in multiple PDAC cohorts and two cohorts of patients treated with immune checkpoint inhibitors, suggesting the TMEscore has the potential to improve precision immunotherapy. The distinct tumor cell-intrinsic and tumor-extrinsic characteristics among tumors with different TMEscores indicate different treatment strategies according to the TMEscore category. Due to the strong intrinsic immunosuppression mechanisms and low cytotoxic infiltration, patients with a low TMEscore are unlikely to benefit from treatment with checkpoint inhibitors alone, but their response to immunotherapy might be rescued through a combined blockade of other intrinsic suppressive molecules, such as TGF signaling or others. Patients with a high TMEscore might benefit more from the immune checkpoint blockage, and their sensitivity to immunotherapy might be enhanced through the association of chemotherapy and/or a STING agonist in order to promote antigen presentation.

## 5. Conclusions

In summary, our study systematically analyzed TME-related genes and proposed a novel TMEscore for risk stratification and selection of PDAC patients in clinical practice. In terms of the future perspectives and implications of our study, it could be useful to further validate the predictive value of the TMEscore in prospective cohorts of patients receiving treatments based on immunotherapy. Furthermore, a comprehensive evaluation of TMEscores with other molecular and genetic markers may provide direction for developing new comprehensive treatment strategies.

## Data Availability

The data presented in this study are contained within the article and Supplementary Materials.

## Supplementary Materials

The following supporting information can be downloaded at: https://www.mdpi.com/article/10.3390/cancers15051442/s1. Supplementary Table S1: Detailed information of retrieved datasets. Supplementary Table S2: TME cell profiles were estimated via the ssGSEA algorithm. Supplementary Table S3: Three robust subtypes of PDAC (named TMEgroups1–3). Supplementary Table S4: Detailed information on the signature genes associated with TMEgroups. Supplementary Table S5: The 74 DEGs for TMEgeneGroup1 and TMEgeneGroup2. Supplementary Table S6: Detailed information of DEGs between TMEgeneGroup1 and TMEgeneGroup2. Supplementary Table S7: The tumor purity for PDAC patients in TCGA. Supplementary Table S8: The feature genes for each mechanism/pathway. Supplementary Table S9: The sequence of oligonucleotides and primers used in this study. Supplementary Table S10: Antibodies used in the experiments. Supplementary Table S11: Univariate and multivariate analysis of overall survival (OS) in PDAC patients. Supplementary Table S12: Univariate and multivariate analysis of disease-free survival (DFS) in PDAC patients. Supplementary Figure S1: A schematic diagram of the process of generation of the TMEscore. Supplementary Figure S2: The filter screening of DEGs. Supplementary Figure S3: GO function and KEGG pathway enrichment analysis of the DEGs between TMEgeneGroup1 and TMEgeneGroup2. Supplementary Figure S4: Prognostic value of TMEscore in six pancreatic cancer cohorts. Supplementary Figure S5: Overexpression of F2RL1 promotes proliferation, migration, and invasion of PDAC cells in vitro.
