# Supplementary material for: Leveraging Tumor Microenvironment Infiltration in Pancreatic Cancer to Identify Gene Signatures Related to Prognosis and Immunotherapy Response
Source: Cancers (Basel). 2023 Feb 24;15(5):1442. doi: 10.3390/cancers15051442 (PMC10000708; doi:10.3390/cancers15051442)
Supplement: Supplementary file 1 [file cancers-15-01442-s001.zip › Supplementary Figures.pdf]

# *Supplementary Figures*

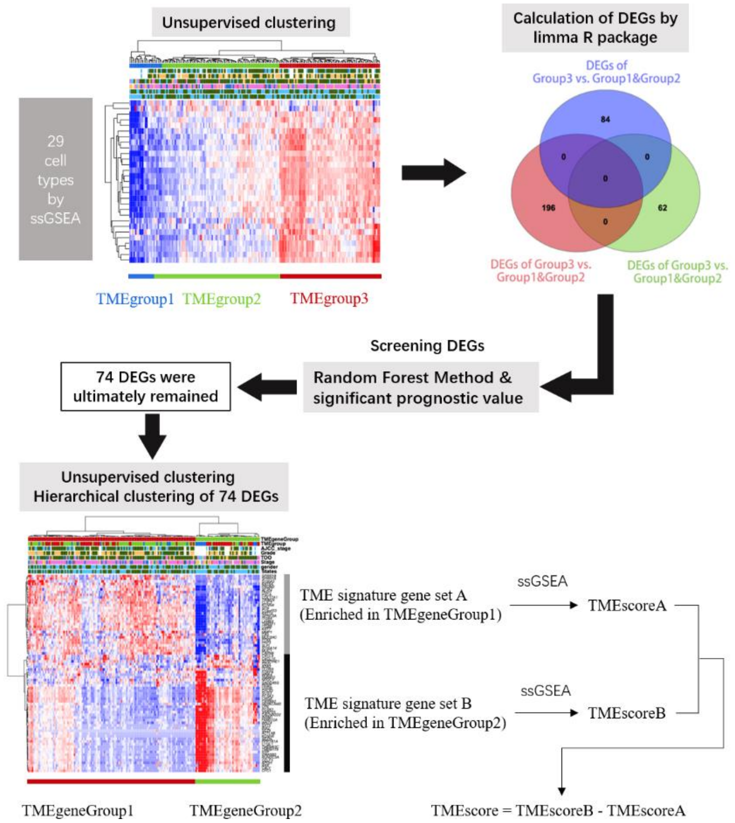


**Supplementary Figure S1.** A schematic diagram of the process of generation of the TMEscore.

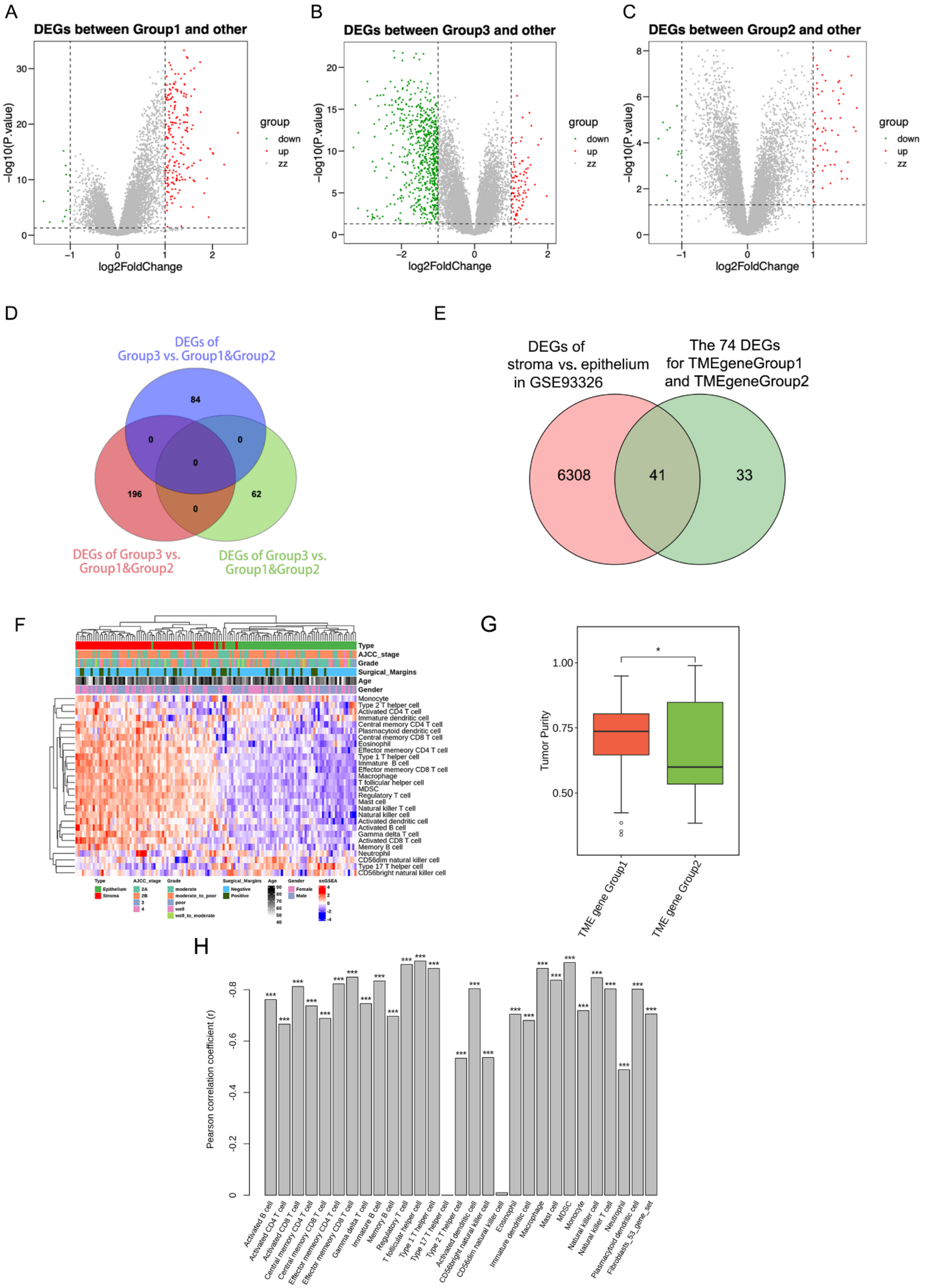
A
DEGs between Group1 and other
-log10(P.value)
log2FoldChange
group
down
up
zz
B
DEGs between Group3 and other
C
DEGs between Group2 and other
D
DEGs of Group3 vs. Group1&Group2
84
0
0
0
196
0
62
DEGs of Group3 vs. Group1&Group2
DEGs of Group3 vs. Group1&Group2
E
DEGs of stroma vs. epithelium in GSE93326
The 74 DEGs for TMEgeneGroup1 and TMEgeneGroup2
6308
41
33
F
Type
AJCC_stage
Grade
Surgical_Margins
Age
Gender
G
Tumor Purity
TME gene Group1
TME gene Group2
H
Pearson correlation coefficient (r)

**Supplementary Figure S2.** The filter screening of DEGs. (A-C) Volcano plots showing the DEGs between a TMEgroup with two other TMEgroups. The detailed information of DEGs was summarized in the Supplementary Table S4. (D)Venn diagram analysis revealed that unique DEGs appeared in all three comparisons. (E) Venn diagram shows the difference between 74 DEGs of TMEgeneGroup and DEGs in GSE93326. (F) Immune cells differences between epithelium and stroma in GSE93326. (G) Tumor purity analysis between TMEgeneGroup1 and TMEgeneGroup2. (H) The bar chart shows the Pearson's correlation coefficient between 29 cell types and tumor purity.

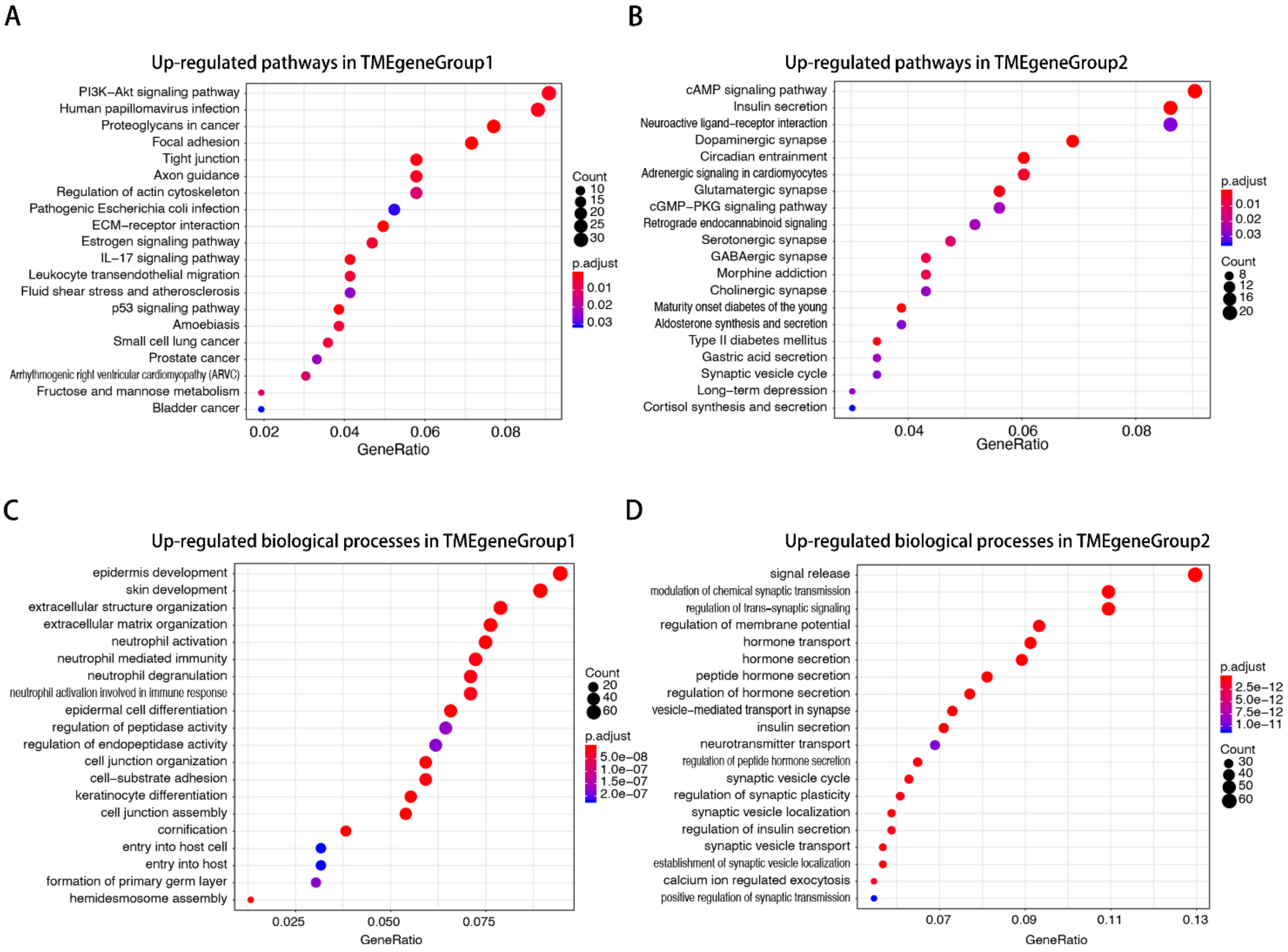


**Supplementary Figure S3.** GO function and KEGG pathway enrichment analysis of the DEGs between TMEgeneGroup1 and TMEgeneGroup2.

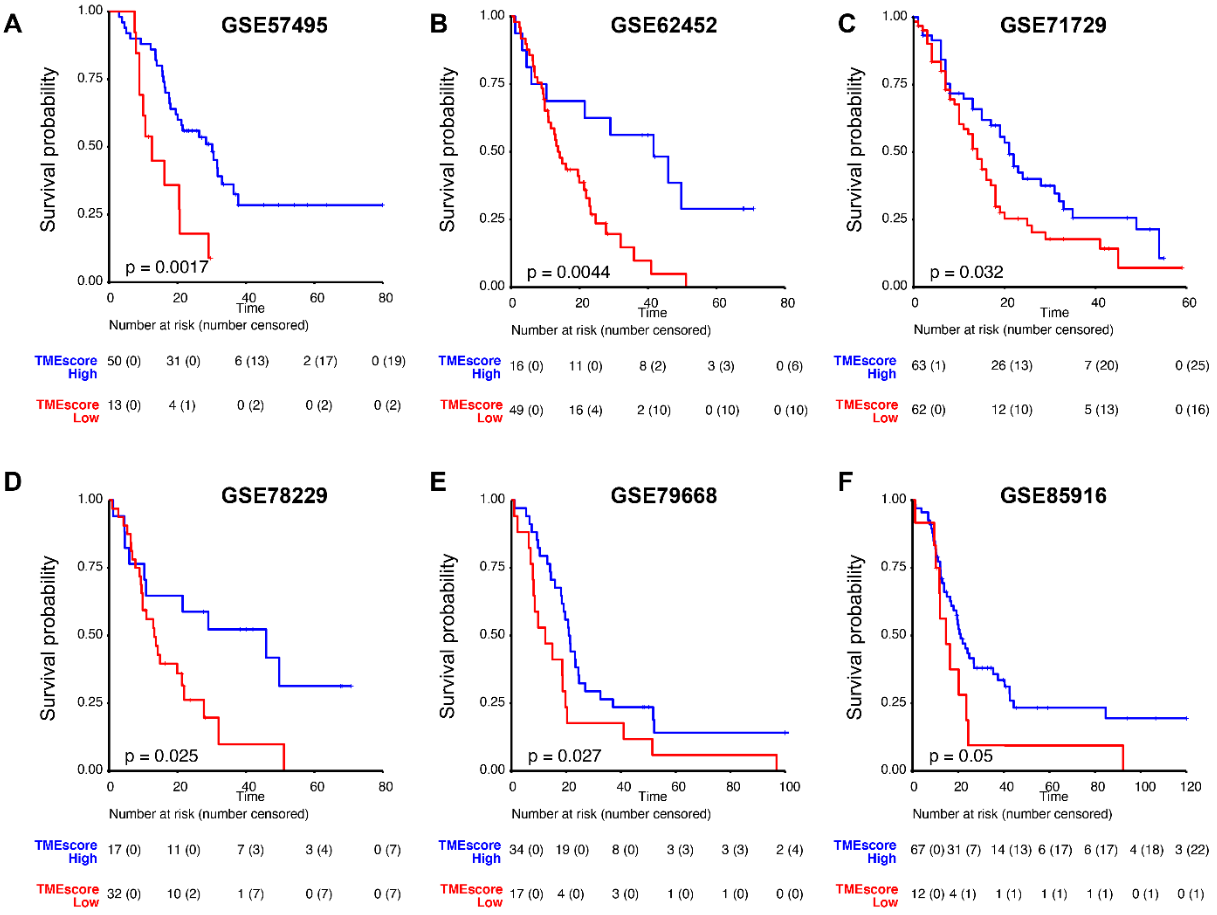


**Supplementary Figure S4.** Prognostic value of TMEscore in six pancreatic cancer cohorts.

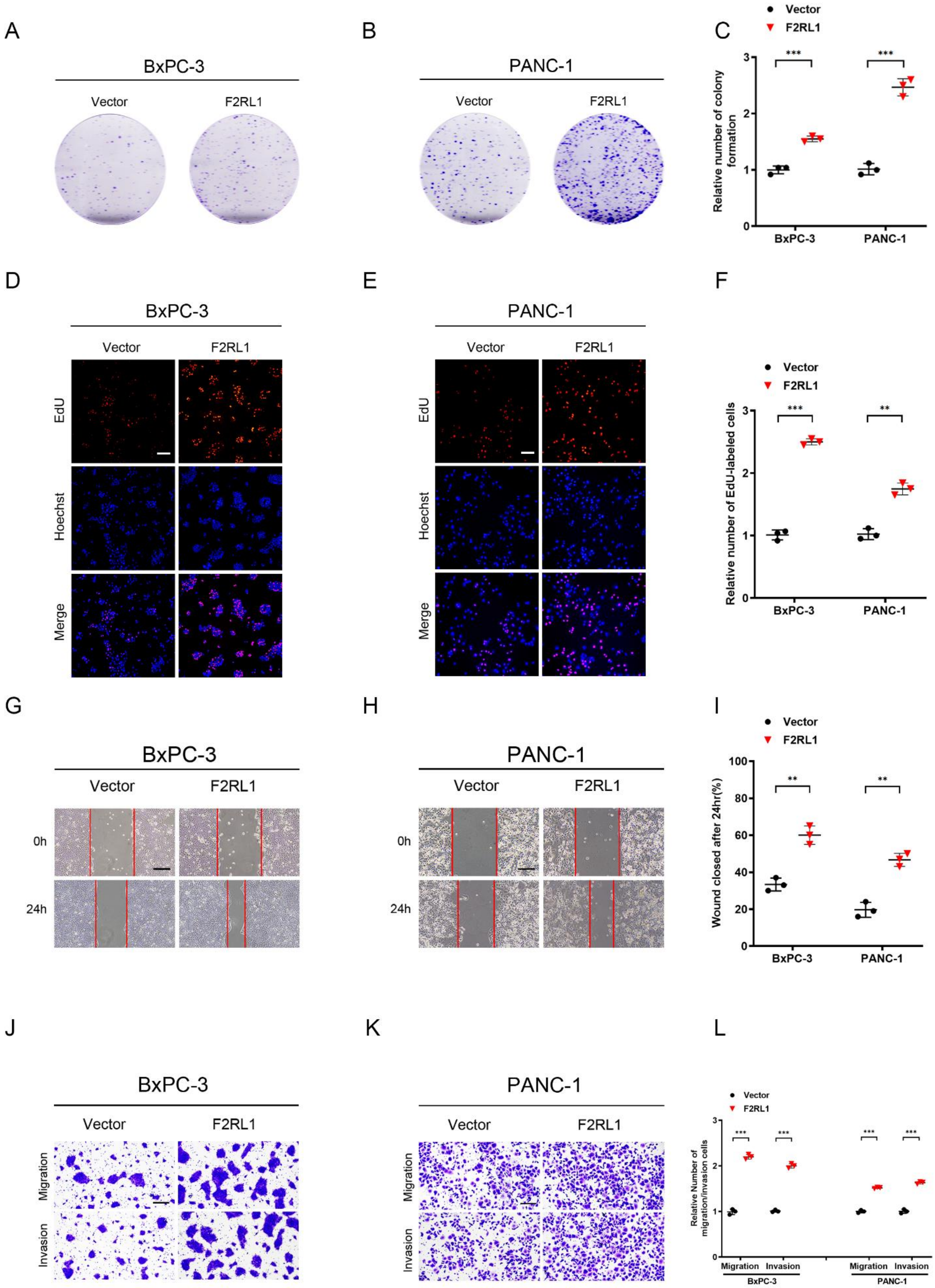
A
BxPC-3
Vector
F2RL1
B
PANC-1
Vector
F2RL1
C
Vector
F2RL1
Relative number of colony formation
BxPC-3
PANC-1
D
BxPC-3
Vector
F2RL1
EdU
Hoechst
Merge
E
PANC-1
Vector
F2RL1
EdU
Hoechst
Merge
F
Vector
F2RL1
Relative number of EdU-labeled cells
BxPC-3
PANC-1
G
BxPC-3
Vector
F2RL1
0h
24h
H
PANC-1
Vector
F2RL1
0h
24h
I
Vector
F2RL1
Wound closed after 24hr(%)
BxPC-3
PANC-1
J
BxPC-3
Vector
F2RL1
Migration
Invasion
K
PANC-1
Vector
F2RL1
Migration
Invasion
L
Vector
F2RL1
Relative Number of migration/invasion cells
Migration
Invasion
BxPC-3
PANC-1

**Supplementary Figure S5.** Overexpression of F2RL1 promotes proliferation, migration and invasion of PDAC cells in vitro. (A-C) The colony formation assay showing representative images (A, B) and quantitative analysis (C) of BxPC-3 and PANC-1 after overexpression of F2RL1. (D-F) EdU assay showing representative images (D, E) and quantitative analysis (F) of BxPC-3 and PANC-1 after overexpression of F2RL1. Scale bars: 100 μm. (G-I) Wound healing assay showing representative images (G, H) and quantitative analysis (I) of BxPC-3 and PANC-1 after overexpression of F2RL1. Scale bars: 100 μm. (J-L) Transwell assay showing representative images (J, K) and quantitative analysis (L) of BxPC-3 and PANC-1 after overexpression of F2RL1. Scale bars: 100 μm. Error bars represent the standard deviations from three independent experiments. (**: $p < 0.01$, ***: $p < 0.001$)
